# Increased Infiltration of CD4 ^+^, CD8 ^+^, and CD68 ^+^ Cells at the Invasive Front Is Associated With Favorable Prognosis in Obstructive Colorectal Cancer: A Retrospective Observational Study

**DOI:** 10.1002/cnr2.70508

**Published:** 2026-03-06

**Authors:** Goro Takahashi, Seiichi Shinji, Toshiyuki Ishiwata, Takeshi Yamada, Kay Uehara, Akihisa Matsuda, Tomio Arai, Ryuji Ohashi, Yasuyuki Yokoyama, Takuma Iwai, Hiroshi Yoshida

**Affiliations:** ^1^ Department of Gastroenterological Surgery Nippon Medical School Tokyo Japan; ^2^ Division of Aging and Carcinogenesis, Research Team for Geriatric Pathology Tokyo Metropolitan Institute for Geriatrics and Gerontology Tokyo Japan; ^3^ Department of Pathology Tokyo Metropolitan Institute for Geriatrics and Gerontology Tokyo Japan; ^4^ Department of Diagnostic Pathology Nippon Medical School Tokyo Japan

**Keywords:** immunofluorescence, obstructive colorectal cancer, tumor microenvironment, tumor‐associated macrophages, tumor‐infiltrating lymphocytes

## Abstract

**Background:**

Obstructive colorectal cancer (OCRC) presents as an oncologic emergency with poor prognosis. Although tumor‐infiltrating lymphocytes (TILs) and tumor‐associated macrophages (TAMs) are known to affect colorectal cancer outcomes, their roles in OCRC remain unclear.

**Methods and Results:**

We retrospectively analyzed 66 patients with Stages II–III OCRC who underwent curative resection following endoscopic decompression. CD4^+^, CD8^+^, and CD68^+^ cell densities at the tumor center and invasive front were quantified using multiplex immunofluorescence imaging. Cancer‐specific survival (CSS) was assessed in relation to immune cell infiltration. Target immune cells were significantly more abundant at the invasive front compared to the tumor center. High densities of CD4^+^ TILs and CD68^+^ TAMs at the invasive front were associated with superior CSS (*p* = 0.0079 and *p* = 0.0088, respectively). Total immune cell density—defined as the sum of CD4^+^, CD8^+^, and CD68^+^ cells/mm^2^—was the strongest independent prognostic factor (HR = 30.8, *p* < 0.001).

**Conclusions:**

High immune cell infiltration at the invasive front was associated with favorable prognosis, even in OCRC. As the invasive front represents the interface between host and tumor, assessment of the tumor immune microenvironment at this site may refine risk stratification and optimize clinical management in OCRC patients.

## 1 Introduction

Colorectal cancer (CRC) is the third most common malignancy and the second leading cause of cancer‐related mortality [1]. Among them, obstructive CRC (OCRC) occurs in approximately 10% of patients with CRC and is an oncological emergency that, if inadequately treated, can lead to electrolyte abnormalities, bacterial translocation, colon perforation, and, in the worst cases, death [2, 3, 4]. Therefore, the ideal clinical management of OCRC involves endoscopic decompression, commonly through the use of a self‐expanding metal stent (SEMS) or a transanal decompression tube (TDT), to stabilize the patient's condition, followed by primary tumor resection [4, 5].

Bowel obstruction is widely recognized as a poor prognostic factor in CRC patients [6, 7, 8]. Both the National Comprehensive Cancer Network and the European Society for Medical Oncology guidelines recommend adjuvant chemotherapy for patients with Stage II OCRC, as well as Stage III, because of a high risk of recurrence [9, 10]. Additionally, studies have shown that obstructive Stage II CRC is associated with worse long‐term outcomes compared with nonobstructive Stage III CRC, emphasizing the severe impact of obstruction on prognosis [8]. This poor prognosis can be influenced by several factors, including advanced tumor depth (T4), malnutrition, and older age [8, 11]. However, the detailed mechanisms underlying these poor outcomes in OCRC remain unclear. Given the impact of bowel obstruction on CRC prognosis, exploring the tumor microenvironment (TME) may be key to understanding these mechanisms and developing better prognostic factors and treatments.

The TME, particularly, immune components such as tumor‐infiltrating lymphocytes (TILs) and tumor‐associated macrophages (TAMs), plays a crucial role in various cancer progression and patient survival [12, 13, 14, 15, 16]. In CRC patients, elevated levels of TILs, such as CD4^+^ T cells and CD8^+^ T cells, have been associated with improved overall survival and disease‐free survival [17, 18, 19]. Similarly, CD68^+^ TAMs correlate with favorable survival in CRC patients [15, 16]. However, data regarding the immune landscape of OCRC, especially at the invasive front—the critical interface of tumor invasion—are lacking.

In this study, we used multiplex immunofluorescence imaging to accurately measure the number of CD4^+^ TILs, CD8^+^ TILs, and CD68^+^ TAMs present in the same tissue sections of OCRC patients. We also evaluated the relationship between the number of these immune cells and clinicopathological features and cancer‐specific survival (CSS).

## 2 Methods

### 2.1 Study Design and Patients

We included consecutive patients with Stage II–III OCRC who underwent primary tumor resection after endoscopic decompression using SEMS or TDT at Nippon Medical School Hospital between January 1, 2010, and March 31, 2021. A diagnosis of OCRC required fulfillment of all the following criteria: severe abdominal symptoms (e.g., abdominal distension and cessation of defecation), marked dilation of the colon requiring decompression on abdominal computed tomography scan, and severe stenosis or obstruction on colonoscopy. The exclusion criteria were as follows: patients who were decompressed with a transnasal long tube, patients who received chemotherapy before primary tumor resection, patients with stent migration, and patients who underwent emergency surgery due to perforation or poor decompression.

### 2.2 Histopathology

Pathological staging was performed according to the Union for International Cancer Control *TNM Classification of Malignant Tumours* (8th edition). Histological type, depth of invasion, lymphatic invasion, vascular invasion, and pathological stage were also obtained from patient pathology reports.

### 2.3 Multiplex Fluorescence Immunohistochemistry and Image Analysis

Specimen sections (5 μm thick) obtained from formalin‐fixed, paraffin‐embedded OCRC tissue were subjected to multiplex immunofluorescence staining using the Opal Multiplex IHC Kit (Akoya Biosciences, Marlborough, MA, US), which allows simultaneous staining of up to seven colors. The antibodies, dilutions, and activation conditions are listed in Table S1. The tissues were stained for key immuno‐oncology markers as follows: CD4 (Opal 650; excitation (ex.) 627 nm, emission (em.) 650 nm), CD8 (Opal 520; ex. 494 nm, em. 525 nm), CD68 (Opal 570; ex. 550 nm, em. 570 nm), cytokeratin (CK, Opal 540; ex. 523 nm, em. 536 nm), alpha‐smooth muscle actin (αSMA, Opal 690; ex. 676 nm, em. 694 nm), and 4′,6‐diamidino‐2‐phenylindole (DAPI), a nuclear counterstain (ex. 358 nm, em. 461 nm) (Figure S1a). Imaging was performed using an automated multispectral system (PhenoImager Mantra2, Akoya Biosciences) that objectively distinguishes cellular signals based on their distinct fluorescent wavelengths within the same tissue section. Fields from the invasive front and the tumor center were manually selected for hotspot analysis based on the highest lymphocyte and macrophage infiltration in the field of view of a ×20 objective lens.

For image analysis, we utilized image analysis software (inForm; AKOYA Biosciences) to process tumor tissue images stained with multiple fluorescent dyes. First, tissue segmentation was conducted to distinguish CK‐positive cancer cell regions from αSMA‐positive stromal areas (Figure S1b). Next, automated cell nucleus recognition was applied (Figure S1c), and phenotyping was subsequently performed by integrating the fluorescence signals from both nucleus and cytoplasmic immunostains (Figure S1d). Finally, these data were merged to quantify the distribution of immune cells (CD4, CD8, CD68) in the stromal area of the same tissue section (Figure S1e). Measurements were taken from three regions, and the average for each type of immune cell was calculated. Preliminary tests for tissue segmentation and phenotype recognition were performed repeatedly until the algorithm reached the level of confidence recommended (at least 90% accuracy) before the final examination [20, 21]. The target cells were defined as follows: CD4^+^ T cells (CD4^+^ TILs): cells positive for DAPI and CD4, CD8^+^ T cells (CD8^+^ TILs): cells positive for DAPI and CD8, CD68^+^ macrophages (CD68^+^ TAMs): cells positive for DAPI and CD68, CRC cells: cells positive for DAPI and CK, and cancer‐associated fibroblasts (CAFs): cells positive for DAPI and αSMA.

## 3 Statistical Analysis

Fisher's exact test and the *χ* ^2^ test were used for comparisons of categorical variables, as appropriate. The Mann–Whitney *U* test was used for comparisons of quantitative data, including the number of target cells. Quantitative data are presented as medians (interquartile ranges; IQR). The number of target cells is presented using box‐and‐whisker plots with median and IQR; the whiskers indicate the 10th and 90th percentile values. CSS was defined as the time from the date of surgery to the date of death due to CRC progression, or the last follow‐up. Using the cutoff values of TILs obtained by receiver operating characteristic (ROC) analysis for cancer specific death, we divided the included patients into two groups (high or low density) and analyzed long‐term prognosis. We used the Kaplan–Meier method for survival curve analysis, the log‐rank test to evaluate differences between groups, and univariate and multivariate Cox proportional hazards regression models to identify prognostic factors for CSS. Significant variables in the univariate analysis (*p* < 0.05) were included in the multivariate Cox regression model. Hazard ratios (HRs) estimated from the Cox regression analysis were reported as relative risks with corresponding 95% confidence intervals (CIs). All statistical analyses were performed using EZR (Saitama Medical Center, Jichi Medical University), a graphical user interface for R (ver. 3.0.2; R Foundation for Statistical Computing, Vienna, Austria) software. All analyses were two‐sided, and differences for which *p* < 0.05 were considered significant.

## 4 Results

### 4.1 Patient Characteristics

**FIGURE 1:**
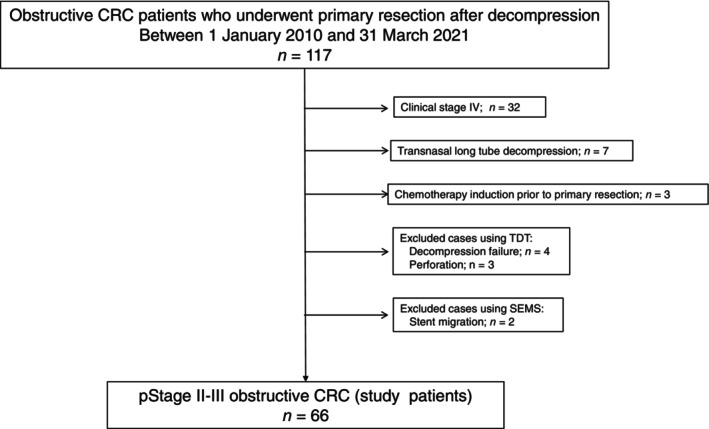
Flow diagram of patient inclusion. CRC, colorectal cancer; pStage, pathological stage; SEMS, self‐expanding metallic stent; TDT, transanal decompression tube.

Among the 117 patients with OCRC, 51 patients were excluded owing to disease at clinical Stage IV (*n* = 32), decompression with a transnasal long tube (*n* = 7), chemotherapy induction before primary tumor resection (*n* = 3), decompression failure with TDT (*n* = 4), emergency surgery because of perforation after TDT insertion (*n* = 3), and stent migration (*n* = 2). As a result, 66 patients with OCRC were included in this study (Figure 1). The patient characteristics are summarized in Table 1. Among the 66 patients, 33 had their obstruction decompressed using SEMS and 33 using TDT. The median interval from decompression to elective surgery was 25 days for SEMS and 9 days for TDT. The distribution of pathological stages was as follows: Stage II, 28 and Stage III, 38 patients. Postoperative chemotherapy was administered in 30 patients (45.4%). The median follow‐up period was 40.0 months (IQR: 23.5–61.2 months). Fifteen patients (22.7%) died during the follow‐up period due to cancer progression.

**TABLE 1:** Patient characteristics.

	Total (*n* = 66)
Age, years	76 [66–82]
Sex, male (%)	42 (63.6)
Ethnicity, Asian (%)	66 (100)
Decompression device (%)	
SEMS	33 (50)
TDT	33 (50)
Time from device placement to surgery, days	
SEMS	25 [20–32]
TDT	9 [6–11]
Tumor location (%)	
Right	18 (27.2)
Left	48 (72.8)
Tumor differentiation (%)	
tub1, tub2	60 (90.9)
por, muc, pap	6 (9.1)
Depth of invasion (%)	
T3	42 (63.6)
T4	24 (36.4)
Lymphatic invasion (%)	
0, 1a	46 (69.7)
1b, 1c	20 (30.3)
Vascular invasion (%)	
0, 1a	41 (62.1)
1b, 1c	25 (37.9)
Pathological TNM stage^a^ (%)	
II	28 (42.4)
III	38 (57.6)
Preoperative CEA level, ng/mL	5.1 [2.9–12.3]
Postoperative chemotherapy (%)	30 (45.4)

*Note:* Data are presented as numbers (%) or medians [interquartile ranges].

Abbreviations: CEA, carcinoembryonic antigen; SEMS, self‐expanding metallic stent; TDT, transanal decompression tube; TNM, tumor node metastasis.

^a^ Union for International Cancer Control *TNM Classification of Malignant Tumours*, 8th edition.

### 4.2 TIL and TAM Densities at the Invasive Front and Tumor Center

**FIGURE 2:**
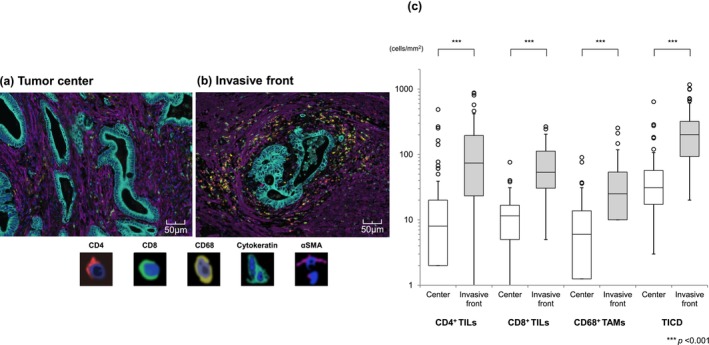
Comparison of TIL and TAM density between the tumor center and the invasive front. Representative multispectral images of CD4^+^ and CD8^+^ TILs, CD68^+^ TAMs, cytokeratin‐positive tumor cells, and αSMA‐positive cancer‐associated fibroblasts are shown for the tumor center (a) and the invasive front (b). (c) Quantitative image analysis demonstrated that immune cell densities were consistently higher at the invasive front than in the tumor center: CD4^+^ TILs, 74 versus 8.0 cells/mm^2^ (*p* < 0.001); CD8^+^ TILs, 53 versus 11 cells/mm^2^ (*p* < 0.001); and CD68^+^ TAMs, 25 versus 6.0 cells/mm^2^ (*p* < 0.001). Overall, the TICD, calculated as the sum of CD4^+^, CD8^+^, and CD68^+^ cell densities, was significantly higher at the invasive front than the tumor center (201 vs. 31 cells/mm^2^, *p* < 0.001). CD, cluster of differentiation; TAM, tumor‐associated macrophage; TICD, total immune cell density; TIL, tumor‐infiltrating lymphocyte; αSMA, alpha‐smooth muscle actin.

Representative multiplex immunofluorescence images of the tumor center and invasive front, accompanied by quantitative analyses of TILs and TAMs in both regions, are presented in Figure 2. The densities of all three types of immune cells in the stroma were significantly higher at the invasive front compared to the tumor center: CD4^+^ TILs, 74 versus 8.0 cells/mm^2^ (*p* < 0.001); CD8^+^ TILs, 53 versus 11 cells/mm^2^ (*p* < 0.001); and CD68^+^ TAMs, 25 versus 6.0 cells/mm^2^ (*p* < 0.001). Total immune cell density (TICD) (sum of CD4^+^, CD8^+^, and CD68^+^ cells) was also significantly higher at the invasive front than in the tumor center (201 versus 31 cells/mm^2^, *p* < 0.001).

### 4.3 CSS Analysis According to the Density of TILs and TAMs in the Tumor Center and at the Invasive Front

**FIGURE 3:**
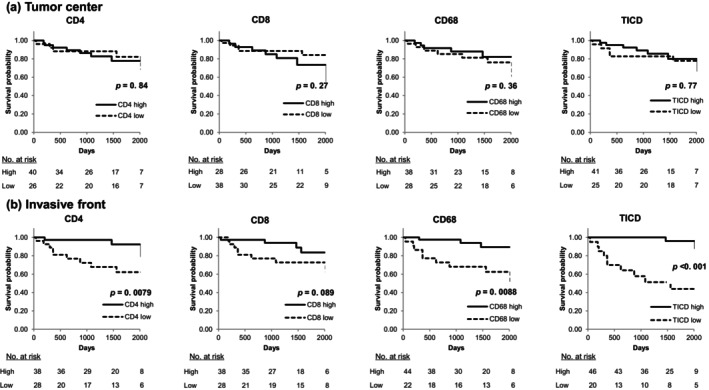
Cancer‐specific survival according to TIL and TAM densities. Kaplan–Meier analyses were performed using image‐based quantitative measurements from 66 patients. High and low groups for CD4^+^ TIL, CD8^+^ TIL, CD68^+^ TAM, and TICD were defined using ROC‐based cut‐off values for cancer‐specific death. (a) Tumor center: Stratification using these cut‐off values showed no significant differences in CSS between high‐ and low‐density groups. (b) Invasive front: High densities of CD4^+^ TILs, CD68^+^ TAMs, and TICD were significantly associated with better CSS (CD4^+^ TIL, *p* = 0.0079; CD68^+^ TAM, *p* = 0.0088; TICD, *p* < 0.001). Among these markers, TICD demonstrated the strongest association with CSS. CD, cluster of differentiation; CSS, cancer‐specific survival; ROC, receiver operating characteristic; TAM, tumor‐associated macrophage; TICD, total immune cell density; TIL, tumor‐infiltrating lymphocytes.

Next, we examined the relationship between the densities of immune cells in the tumor center or invasive front area and CSS. In the tumor center, the cutoff values obtained through ROC analysis used to divide patients into two groups were as follows: CD4^+^ TILs, 4.0 cells/mm^2^; CD8^+^ TILs, 12 cells/mm^2^; CD68^+^ TAMs, 3.0 cells/mm^2^; and TICD, 21 cells/mm^2^ (Table S2). Stratification based on the density of TILs and TAMs in the tumor center showed no statistically significant differences between the two groups in relation to CSS (*p* = 0.84, *p* = 0.27, *p* = 0.36, and *p* = 0.77, respectively) (Figure 3a).

At the invasive front, the cutoff values obtained through ROC analysis to divide patients into two groups were as follows: CD4^+^ TILs, 48 cells/mm^2^; CD8^+^ TILs, 45 cells/mm^2^; CD68^+^ TAMs, 14 cells/mm^2^; and TICD, 116 cells/mm^2^ (Table S2). High densities of CD4^+^ TILs, CD68^+^ TAMs, and TICD were significantly associated with improved CSS (*p* = 0.0079, *p* = 0.0088, and *p* < 0.001, respectively) (Figure 3b). Additionally, a high density of CD8^+^ TILs at the invasive front showed a trend toward better prognosis, although this association did not reach statistical significance (*p* = 0.089).

### 4.4 Univariate and Multivariate Cox Regression Analyses for CSS

Univariate and multivariate analyses for CSS were performed using prognostic factors including age, sex, tumor invasion depth, lymphatic and venous invasion, pathological stage, preoperative carcinoembryonic antigen level, postoperative chemotherapy induction, and TICD at the invasive front (Table 2). Univariate analysis showed that severe lymphatic invasion and TICD at the invasive front were associated with CSS (HR: 3.16, 95% CI: 1.13–8.81, *p* = 0.027, and HR: 11.4, 95% CI: 3.14–40.8, *p* < 0.001, respectively). Multivariate analysis confirmed that severe lymphatic invasion and TICD at the invasive front were independent prognostic factors for CSS (HR: 12.7, 95% CI: 3.01–53.5, *p* < 0.001, and HR: 30.8, 95% CI: 6.39–148, *p* < 0.001, respectively).

**TABLE 2:** Univariate and multivariate Cox regression analyses for cancer‐specific survival.

Variables	Univariate analysis			Multivariate analysis		
	HR	95% CI	*p*	HR	95% CI	*p*
Age (≥ 75 years)	2.07	0.71–5.97	0.17			
Sex (male vs. female)	1.73	0.55–5.4	0.34			
Tumor invasion depth (T4 vs. T3)	1.21	0.43–3.43	0.71			
Lymphatic invasion (ly1b, 1c vs. ly0, 1a)	3.16	1.13–8.81	0.027	12.7	3.01–53.5	< 0.001
Venous invasion (v1b, 1c vs. v0, 1a)	1.63	0.68–3.94	0.27			
Pathological stage (Stages III vs. II)	1.52	0.51–4.46	0.44			
Preoperative CEA level (> 5.0 vs. ≦ 5.0 ng/mL)	1.70	0.61–4.70	0.30			
Postoperative chemotherapy (yes vs. no)	0.42	0.13–1.34	0.14			
TICD at invasive front^a^ (low‐ vs. high‐density)	11.4	3.14–40.8	< 0.001	30.8	6.39–148	< 0.001

Abbreviations: CEA, carcinoembryonic antigen; CI, confidence interval; CSS, cancer‐specific survival; HR, hazard ratio; TICD, total immune cell density.

^a^ Total target cell density is the sum of CD4^+^, CD8^+^, and CD68^+^ cells.

### 4.5 Association Between Clinicopathological Findings and TICD

The association between clinicopathological findings and TICD at the invasive front is shown in Table 3. When comparing the low‐TICD group (*n* = 20) to the high‐TICD group (*n* = 46), a statistically significant difference was observed in the neutrophil‐to‐lymphocyte ratio (NLR) (*p* = 0.035). Specifically, the low‐TICD group exhibited higher NLR values (3.64 [2.48–6.07]) than the high‐TICD group (2.67 [2.05–3.84]). In addition, the prognostic nutritional index (PNI) tended to be higher in the high‐TICD group (*p* = 0.073). Other factors, such as tumor invasion depth, tumor size, tumor differentiation, lymphatic and vascular invasion, and pathological stage, did not statistically differ between the two groups.

**TABLE 3:** Association between patients' background factors and total immune cell density.

Variables	TICD at the invasive front		*p*
	Low group (*n* = 20)	High group (*n* = 46)	
Tumor invasion depth			
T3	9	33	0.052
T4	11	13	
Tumor size			
< 55 mm	5	22	0.10
≥ 55 mm	15	24	
Tumor differentiation			
well, mod	16	43	0.18
por, muc, pap	4	3	
Lymphatic invasion			
High (ly1b, 1c)	10	17	0.77
Low (ly0, 1a)	10	29	
Vascular invasion			
High (v1b, 1c)	9	19	0.41
Low (v0, 1a)	11	27	
Pathological stage			
II	9	19	0.67
III	11	27	
Neutrophil‐to‐lymphocyte ratio	3.64 [2.48–6.07]	2.67 [2.05–3.84]	0.035
Prognostic nutritional index	38.3 [33.9–42.7]	42.9 [37.6–46.3]	0.073

Abbreviations: mod, moderately differentiated; muc, mucinous; pap, papillary; por, poorly differentiated; TICD, total immune cell density; well, well differentiated.

### 4.6 Association Between the Density of TILs and TAMs at the Invasive Front and the Type of Decompression Device

**FIGURE 4:**
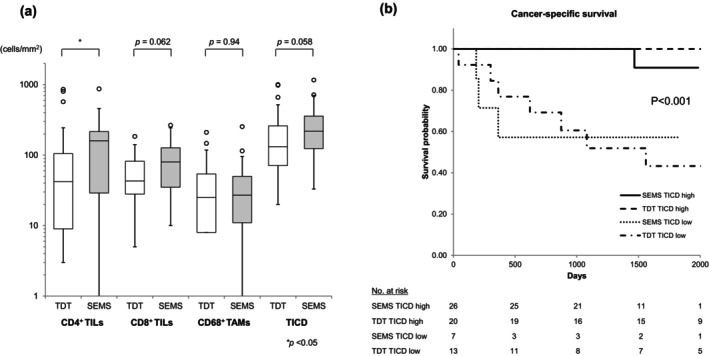
Comparison of TIL and TAM density at the invasive front between TDT and SEMS. (a) Tumors decompressed using SEMS exhibited significantly higher CD4^+^ TIL density compared to those treated with TDT (160 vs. 42 cells/mm^2^, *p* = 0.033). CD8^+^ TIL density (80 vs. 43 cells/mm^2^, *p* = 0.058) and TICD (218 vs. 132 cells/mm^2^, *p* = 0.062) also showed a trend toward higher values in the SEMS group. CD68^+^ TAM density was comparable between the two methods (27 vs. 26 cells/mm^2^, *p* = 0.94). (b) A higher proportion of TICD‐high cases was observed in the SEMS group than in the TDT group (78.7% vs. 60.6%, *p* = 0.18). Survival outcomes, however, were similar between SEMS and TDT in both the TICD‐high and TICD‐low subgroups. CD, cluster of differentiation; SEMS, self‐expanding metallic stent; TAM, tumor‐associated macrophage; TDT, transanal decompression tube; TICD, total immune cell density; TIL, tumor‐infiltrating lymphocyte.

Tumors decompressed with SEMS exhibited a significantly higher CD4^+^ TIL density than those treated with TDT (160 vs. 42 cells/mm^2^, *p* = 0.033) (Figure 4a). CD8^+^ TIL density (80 vs. 43 cells/mm^2^, *p* = 0.058) and TICD (218 vs. 132 cells/mm^2^, *p* = 0.062) also showed higher tendencies in the SEMS group, though these differences were not statistically significant. In contrast, CD68^+^ TAM density showed no difference between the two devices (27 vs. 26 cells/mm^2^, *p* = 0.94). When patients were stratified using the ROC‐derived TICD cutoff, the proportion of TICD‐high cases was numerically higher in the SEMS group (78.7% vs. 60.6%, *p* = 0.18), although this difference was not statistically significant. Within both TICD‐high and TICD‐low groups, survival outcomes were similar between SEMS and TDT, with no observable prognostic differences between devices (Figure 4b).

## 5 Discussion

This study is the first to comprehensively evaluate TILs and TAMs in OCRC using multiplex immunofluorescence. We demonstrated that immune cell densities—particularly at the invasive front—are strongly associated with long‐term prognosis in OCRC patients.

Previous studies have extensively characterized the tumor immune microenvironment in CRC [12, 13, 14, 15, 16], including diverse T‐cell phenotypes such as regulatory and exhausted T cells [22, 23] and macrophage polarization (M1/M2) [24]. These immune features have been shown to correlate with prognosis and therapeutic responses in conventional CRC. In contrast, OCRC has remained virtually unexamined from an immunological perspective, despite its distinct clinical presentation and poorer outcomes. Whether immune characteristics identified in nonobstructive CRC cohorts are preserved or altered in OCRC has remained largely unclear.

To address these uncertainties, we first examined the spatial distribution of key immune cell populations within OCRC tissues. The densities of CD4^+^ TILs, CD8^+^ TILs, and CD68^+^ TAMs at the invasive front were 9.3‐, 4.8‐, and 4.2‐fold higher, respectively, than those in the tumor center. Notably, this disparity was far greater than that typically observed in conventional CRC, where the invasive front usually shows only a modest 1.7–2.0‐fold increase in TIL density relative to the tumor center [25, 26]. This distribution pattern is consistent with a “cold tumor” phenotype characterized by immune exclusion from the tumor core [27, 28]. In OCRC, CAF‐driven stromal remodeling may be more extensive than in conventional CRC, generating dense extracellular matrices that restrict immune cell infiltration [29]. These findings indicate that immune infiltration in OCRC is not broadly distributed throughout the tumor mass but is instead largely confined to the invasive front. Based on this observation, we next evaluated whether TIL and TAM densities at the invasive front carried prognostic significance.

We then found that high CD4^+^ TILs, CD68^+^ TAMs, and TICD at the invasive front were associated with superior CSS in OCRC patients. In multivariate analysis, low TICD emerged as the most powerful prognostic factor (HR 30.8, 95% CI 6.39–148; *p* < 0.001), surpassing conventional clinicopathological features such as severe lymphatic invasion (HR 12.7). Consistent with previous CRC studies demonstrating that abundant CD4^+^ or CD8^+^ TILs and CD68^+^ TAMs predict favorable outcomes [12, 13, 14, 15, 16], our data underscore the prognostic primacy of the tumor immune contexture (i.e., immune landscape), and suggest that immune depletion at the invasive front can drive poor prognosis of OCRC.

Among the various background factors, the NLR and PNI demonstrated notable associations with TICD at the invasive front. The NLR, which has been previously reported as an indicator of poor prognosis in CRC [30], was significantly higher in patients with low TICD. This finding suggests that a higher systemic inflammatory response can be associated with a lower TICD, potentially reflecting poor antitumor immunity at the invasive front. Similarly, PNI, an indicator of a patient's nutritional status and cancer prognosis [31, 32], also showed a potential association with TICD. Patients with OCRC frequently exhibit systemic inflammation and poor nutritional status, which may compromise antitumor immunity at the invasive front [33]. This highlights the need for interventions to enhance overall patient condition before surgery. Therefore, appropriate preoperative decompression and nutritional support may improve general patient condition, potentially enhancing antitumor immunity.

Interestingly, we observed that patients who underwent SEMS decompression tended to have higher densities of TILs at the invasive front compared with those managed with TDT. A modest trend toward a higher proportion of TICD‐high tumors in the SEMS group was also noted. One plausible explanation is that SEMS, by providing more reliable luminal patency, may support better preoperative nutritional status, attenuate systemic inflammation and malnutrition, and allow more effective management of comorbidities [4]. Each of these factors could contribute to a partial restoration of antitumor immunity. By contrast, the shorter interval to surgery in TDT patients (median 9 vs. 25 days with SEMS; Table 1) may not afford sufficient time for such physiological recovery, leaving them in a more catabolic state with persistently lower TIL levels. While the difference did not reach statistical significance, the higher proportion of TICD‐high cases in the SEMS group represents a novel observation. This trend raises the possibility that SEMS may facilitate recovery of systemic and local immune status through more stable decompression and improved perioperative condition. Although these findings hint that SEMS might serve as a more immunologically favorable bridge to surgery than TDT, our study is limited by its retrospective design and relatively small sample size. Prospective, ideally randomized, investigations will be needed to confirm whether SEMS truly alters the tumor immune microenvironment and translates into improved oncological outcomes.

Multiplex immunofluorescence allowed precise spatial analysis of immune cells while preserving tissue architecture, offering advantages over conventional immunohistochemistry [34]. However, its technical complexity, potential signal overlap, and challenges in data interpretation necessitate careful validation to ensure its clinical applicability. This study was intentionally designed as an initial overview of the immune contexture in OCRC, aiming to delineate the spatial distribution of key immune cell populations and their prognostic relevance. Given the technical constraints of multiplex immunofluorescence—which permits simultaneous detection of up to seven markers per tissue section—we had already allocated six of these available channels to essential markers (CD4, CD8, CD68, CK, αSMA, and DAPI). Expanding the marker panel further would have required additional tissue sections and risked compromising sample size and analytical uniformity. Therefore, we prioritized consistent quantification across all cases. As such, this work represents a first step toward characterizing the immune microenvironment in OCRC.

Taken together, this study provides an initial immunological framework for OCRC and highlights the need to consider obstruction‐associated factors when interpreting tumor–immune interactions in CRC.

Our study has several limitations. First, this study was a single‐center retrospective analysis with a relatively small sample size. As a result, the HRs for TICD and lymphatic invasion were notably high in the multivariate analysis (30.8 and 12.7, respectively), which may have influenced the stability of the multivariate model. Second, given the exploratory nature of this study and the technical constraints of multiplex immunofluorescence, functional characterization of immune cell subsets—such as CD4^+^ and CD8^+^ T‐cell phenotypes (e.g., regulatory or exhausted T cells) [22, 23] and macrophage polarization states (M1/M2) [24]—was beyond the scope of this investigation. Future prospective studies with larger cohorts and expanded immune profiling will be essential to further elucidate antitumor immunity in OCRC.

## 6 Conclusions

In summary, we demonstrated that high densities of TILs and TAMs at the invasive front are associated with improved long‐term prognosis in patients with OCRC. Among these, TICD at the invasive front was identified as an independent prognostic factor, providing greater predictive value than conventional clinicopathological variables. Our findings suggest that evaluating the tumor immune microenvironment—particularly, at the invasive front—may improve prognostic assessment and inform treatment strategies in this challenging clinical subset. Further prospective, multicenter studies are warranted to validate these observations and explore their clinical applicability.

## Author Contributions

**Goro Takahashi:** conceptualization, methodology, data curation, formal analysis, investigation, visualization, writing – original draft, project administration, funding acquisition. **Seiichi Shinji:** validation, writing – review and editing, project administration, investigation, funding acquisition. **Toshiyuki Ishiwata:** investigation, project administration, writing – review and editing. **Takeshi Yamada:** supervision. **Kay Uehara:** supervision. **Akihisa Matsuda:** investigation. **Tomio Arai:** supervision. **Ryuji Ohashi:** supervision. **Yasuyuki Yokoyama:** investigation. **Takuma Iwai:** investigation. **Hiroshi Yoshida:** supervision.

## Funding

This research was funded by Grants‐in‐Aid for Early‐Career Scientists (Grant 19K18165) and Scientific Research (C) (Grant 22K08835) from the Ministry of Education, Culture, Sports, Science, and Technology, Japan, and the Department of Gastroenterological Surgery, Nippon Medical School, respectively.

## Ethics Statement

This retrospective observational study was approved by the Institutional Review Boards of Nippon Medical School (B‐2021‐380) and Tokyo Metropolitan Geriatric Hospital and Institute of Gerontology (R21‐030), and was conducted in accordance with the principles of the Declaration of Helsinki. Owing to the retrospective nature of the study, the requirement for written informed consent was waived.

## Conflicts of Interest

The authors declare no conflicts of interest.

## Supporting information


**Figure S1:** Assessment of tumor microenvironment using. (a) Composite image of a tissue section stained using multiplex fluorescence IHC for CD4 (Opal 650, red), CD8 (Opal 520, green), CD68 (Opal 570, yellow), CK (Opal 540, cyan), and αSMA (Opal 690, purple) along with a DAPI nuclear counterstain. (b) Segmentation distinguishes cancer cell regions (Ca, CK‐positive) from stromal areas (St, αSMA‐positive). (c) Automated cell nucleus recognition. (d) Phenotyping integrating nuclear and cytoplasmic signals. (e) Merged image (b–d) used for quantifying target cells in the stroma. αSMA, alpha‐smooth muscle actin; CAF, cancer‐associated fibroblast; CD, cluster of differentiation; CK, cytokeratin; DAPI, 4′,6‐diamidino‐2‐phenylindole; IHC, immunohistochemistry; TAM, tumor‐associated macrophage; and TIL, tumor‐infiltrating lymphocyte.

**Table S1:** Immunostaining conditions. This table summarizes the multiplex immunofluorescence staining conditions applied in this study, including the staining order, antibody clones, isotypes, vendors, catalog numbers, fluorophores, antigen retrieval conditions, and dilutions for each marker.

**Table S2:** Cutoff values for tumor‐infiltrating lymphocytes and tumor‐associated macrophages determined by receiver operating curve analysis for cancer‐specific survival. This table shows the cutoff values for TILs and TAMs determined by ROC curve analysis for cancer‐specific survival. Cutoff values, AUCs, odds ratios, and *p*‐values are presented separately for the tumor center and invasive front. AUC, area under the curve; ROC, receiver operating characteristic; TAM, tumor‐associated macrophage; TIL, tumor‐infiltrating lymphocyte.

## Data Availability

The data that support the findings of this study are available from the corresponding author upon reasonable request.
