# Supplementary material for: Increased Infiltration of CD4 +, CD8 +, and CD68 + Cells at the Invasive Front Is Associated With Favorable Prognosis in Obstructive Colorectal Cancer: A Retrospective Observational Study
Source: Cancer Rep (Hoboken). 2026 Mar 6;9(3):e70508. doi: 10.1002/cnr2.70508 (PMC12965902; doi:10.1002/cnr2.70508)
Supplement: Supplementary file 1 — Figure S1: Assessment of tumor microenvironment using. (a) Composite image of a tissue section stained using multiplex fluorescence IHC for CD4 (Opal 650, red), CD8 (Opal 520, green), CD68 (Opal 570, yellow), CK (Opal 540, cyan), and αSMA (Opal 690, purple) along with a DAPI nuclear counterstain. (b) Segmentation distinguishes cancer cell regions (Ca, CK‐positive) from stromal areas (St, αSMA‐positive). (c) Automated cell nucleus recognition. (d) Phenotyping integrating nuclear and cytoplasmic signals. (e) Merged image (b–d) used for quantifying target cells in the stroma. αSMA, alpha‐smooth muscle actin; CAF, cancer‐associated fibroblast; CD, cluster of differentiation; CK, cytokeratin; DAPI, 4′,6‐diamidino‐2‐phenylindole; IHC, immunohistochemistry; TAM, tumor‐associated macrophage; and TIL, tumor‐infiltrating lymphocyte. [file CNR2-9-e70508-s003.pdf]

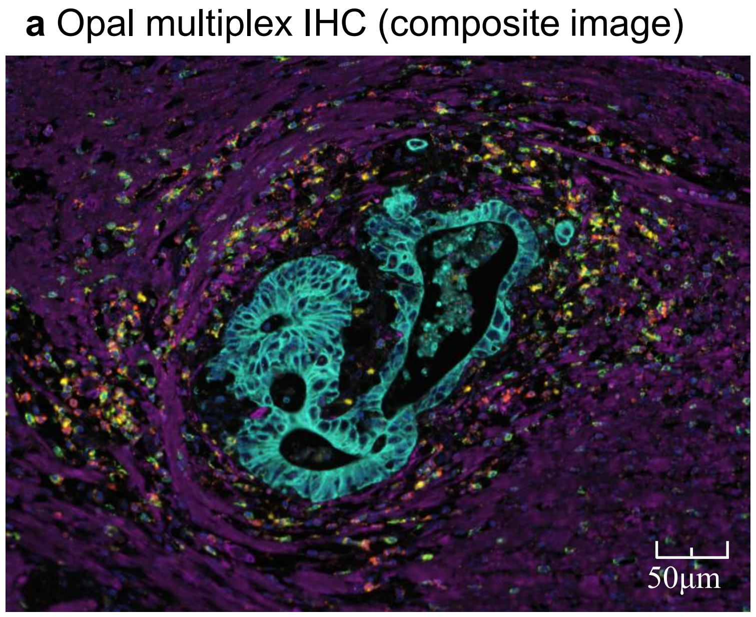

Target	Reagent	Ex (nm)	Em (nm)
CD8	Opal 520	494	525
CK	Opal 540	523	536
CD68	Opal 570	550	570
CD4	Opal 650	627	650
αSMA	Opal 690	676	694

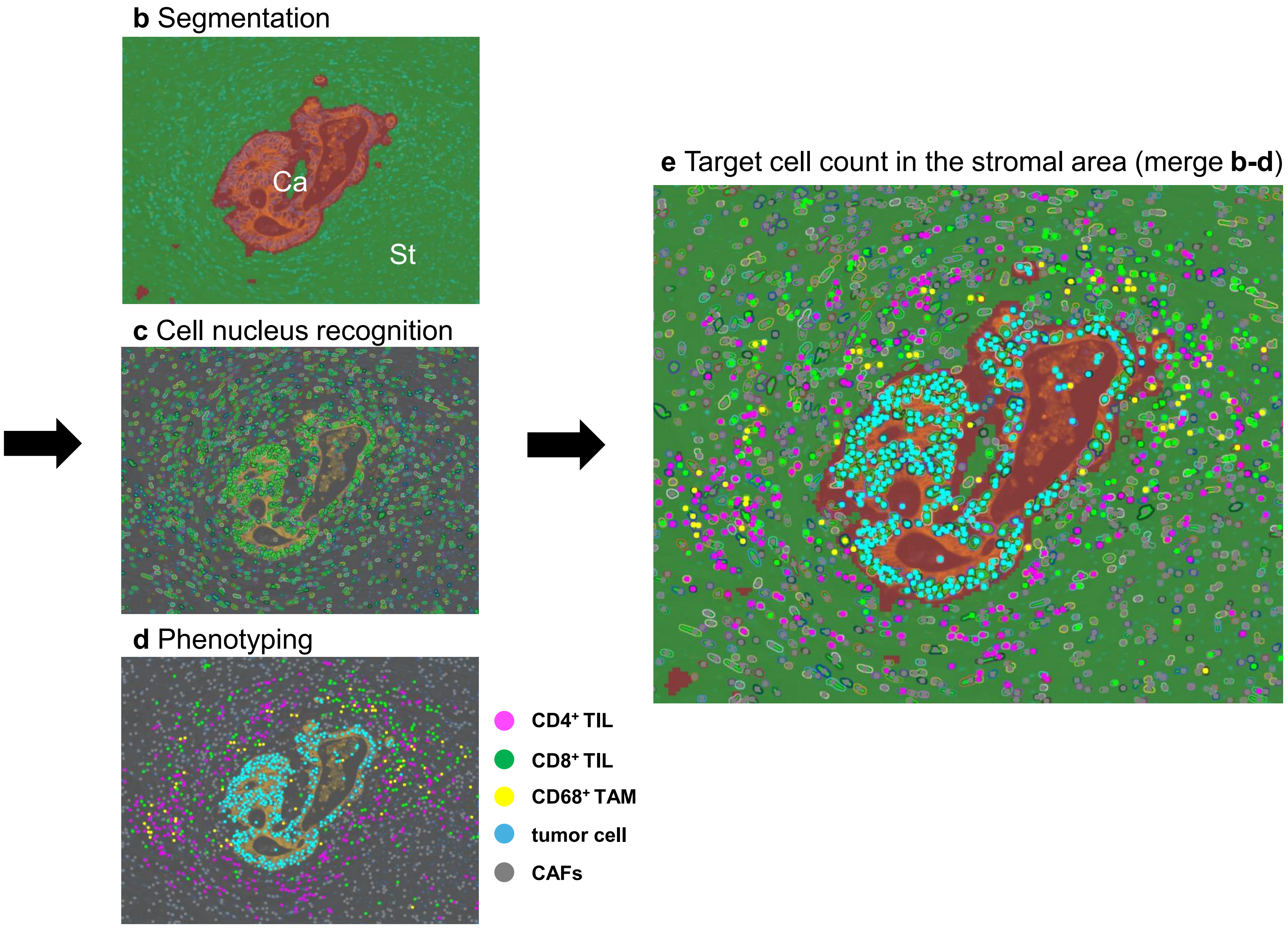

Figure S1 Assessment of tumor microenvironment using multispectral imaging
