# Supplementary material for: Increased Infiltration of CD4 +, CD8 +, and CD68 + Cells at the Invasive Front Is Associated With Favorable Prognosis in Obstructive Colorectal Cancer: A Retrospective Observational Study
Source: Cancer Rep (Hoboken). 2026 Mar 6;9(3):e70508. doi: 10.1002/cnr2.70508 (PMC12965902; doi:10.1002/cnr2.70508)
Supplement: Supplementary file 2 — Table S1: Immunostaining conditions. This table summarizes the multiplex immunofluorescence staining conditions applied in this study, including the staining order, antibody clones, isotypes, vendors, catalog numbers, fluorophores, antigen retrieval conditions, and dilutions for each marker. [file CNR2-9-e70508-s002.docx]

| **Staining order** | **Marker** | **Clone** | **Isotype** | **Company** | **Catalog No.** | **Opal Fluorophore (Ex/Em)** | **Antigen Retrieval** | **Dilution** |
| --- | --- | --- | --- | --- | --- | --- | --- | --- |
| 1st | CD4 | 4B12 | Mouse IgG1 | Leica Biosystems | NCL-L-CD4-368 | Opal 650  (Ex 627 / Em 650 nm) | pH6, 98°C, 15 min | 1:50 |
| 2nd | CD8 | C8/144B | Mouse IgG1 | Abcam | ab4055 | Opal 520  (Ex 494 / Em 525 nm) | pH6, 98°C, 15 min | 1:500 |
| 3rd | CD68 | PG-M1 | Mouse IgG3 | Abcam | ab955 | Opal 570  (Ex 550 / Em 570 nm) | pH6, 98°C, 15 min | 1:100 |
| 4th | Cytokeratin (AE1/AE3) | AE1+AE3 | Mouse IgG1 | Dako | M3515 | Opal 540  (Ex 523 / Em 536 nm) | pH6, 98°C, 15 min | 1:200 |
| 5th | αSMA | 1A4 | Mouse IgG1 | Dako | M0851 | Opal 690  (Ex 676 / Em 694 nm) | pH6, 98°C, 15 min | 1:800 |
| Final | Nuclei | - | - | - | - | DAPI  (Ex 358 / Em 461 nm) | - | - |

Table S1. Detailed Multiplex Immunofluorescence Staining Conditions Including Staining Order
