# Supplementary material for: Increased Infiltration of CD4 +, CD8 +, and CD68 + Cells at the Invasive Front Is Associated With Favorable Prognosis in Obstructive Colorectal Cancer: A Retrospective Observational Study
Source: Cancer Rep (Hoboken). 2026 Mar 6;9(3):e70508. doi: 10.1002/cnr2.70508 (PMC12965902; doi:10.1002/cnr2.70508)
Supplement: Supplementary file 3 — Table S2: Cutoff values for tumor‐infiltrating lymphocytes and tumor‐associated macrophages determined by receiver operating curve analysis for cancer‐specific survival. This table shows the cutoff values for TILs and TAMs determined by ROC curve analysis for cancer‐specific survival. Cutoff values, AUCs, odds ratios, and p‐values are presented separately for the tumor center and invasive front. AUC, area under the curve; ROC, receiver operating characteristic; TAM, tumor‐associated macrophage; TIL, tumor‐infiltrating lymphocyte. [file CNR2-9-e70508-s001.docx]

**Supplementary Table S2** Cutoff values of TILs and TAMs determined by ROC curve analysis for cancer specific survival

A. Tumor center

|  | Cutoff value(/mm^2^) | AUC | Odds ratio | *p* value |
| --- | --- | --- | --- | --- |
| CD4^＋^ TILs | 4.0 | 0.56 | 1.47 | 0.45 |
| CD8^＋^ TILs | 12 | 0.60 | 2.43 | 0.24 |
| CD68^+^ TAMs | 3.0 | 0.70 | 3.66 | 0.010 |
| Total immune cell density | 21 | 0.55 | 1.60 | 0.48 |

B. Invasive front

|  | Cutoff value (/mm^2^) | AUC | Odds ratio | *p* value |
| --- | --- | --- | --- | --- |
| CD4^＋^ TILs | 48 | 0.74 | 5.50 | < 0.001 |
| CD8^＋^ TILs | 45 | 0.68 | 3.66 | 0.0088 |
| CD68^+^ TAMs | 14 | 0.78 | 6.50 | < 0.001 |
| Total immune cell density | 116 | 0.82 | 21.5 | < 0.001 |
